# Phosphoinositides, Major Actors in Membrane Trafficking and Lipid Signaling Pathways

**DOI:** 10.3390/ijms18030634

**Published:** 2017-03-15

**Authors:** Johan-Owen De Craene, Dimitri L. Bertazzi, Séverine Bär, Sylvie Friant

**Affiliations:** Department of Molecular and Cellular Genetics, Université de Strasbourg, CNRS, GMGM UMR 7156, F-67000 Strasbourg, France; dimitri.bertazzi@gmail.com (D.L.B.); sbar@unistra.fr (S.B.)

**Keywords:** lipids, membrane trafficking, vesicles, phosphoinositides, phosphatase, kinase

## Abstract

Phosphoinositides are lipids involved in the vesicular transport of proteins and lipids between the different compartments of eukaryotic cells. They act by recruiting and/or activating effector proteins and thus are involved in regulating various cellular functions, such as vesicular budding, membrane fusion and cytoskeleton dynamics. Although detected in small concentrations in membranes, their role is essential to cell function, since imbalance in their concentrations is a hallmark of many cancers. Their synthesis involves phosphorylating/dephosphorylating positions D3, D4 and/or D5 of their inositol ring by specific lipid kinases and phosphatases. This process is tightly regulated and specific to the different intracellular membranes. Most enzymes involved in phosphoinositide synthesis are conserved between yeast and human, and their loss of function leads to severe diseases (cancer, myopathy, neuropathy and ciliopathy).

## 1. Phosphoinositides in Cellular Membranes

### 1.1. Lipids, Major Membrane Components

The dynamic modulation of the physicochemical properties of membranes is required for eukaryotic cells function. Indeed, cells live in an environment characterized by temperature, relative humidity, pH, sun exposure, osmotic pressure and nutrient variations. Living organisms have to adapt to variations of these different factors in order to keep their intracellular balance. Eukaryotic cells have achieved this by adopting a compartmentalized organization, which minimizes the intracellular variations resulting from extracellular fluctuations. The plasma membrane is the first barrier separating the cytoplasm from the extracellular medium. Its composition ensures a mechanical protection, but also allows exchanges with the medium through transporters and receptors, as a form of very selective permeability.

**Figure 1:**
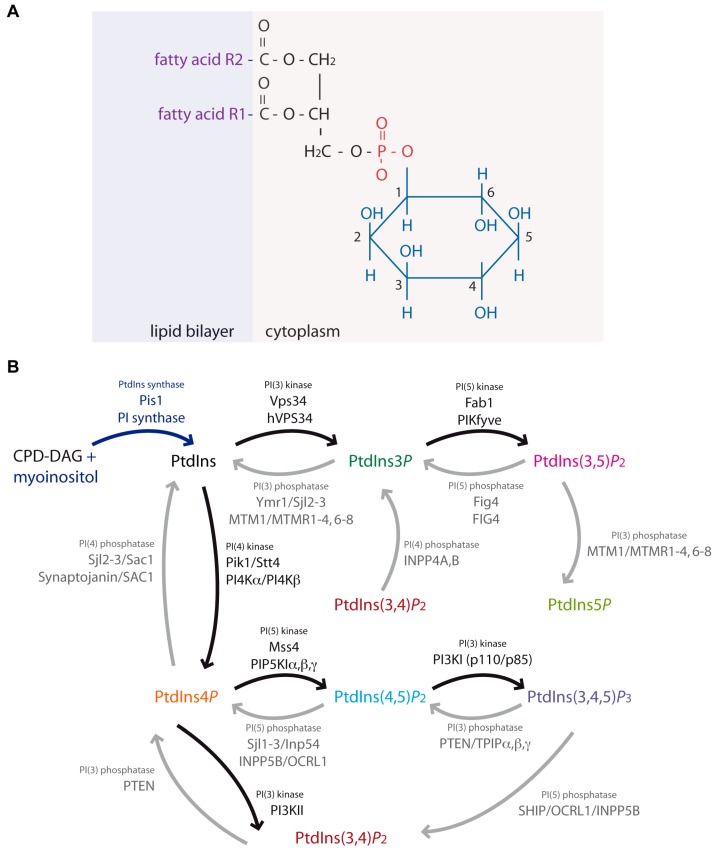
Phosphoinositides synthesized in yeast *Saccharomyces cerevisiae (S. cerevisiae)* and in human cells with the enzymes involved. (**A**) The chemical structure of phosphatidylinositol (PI); (**B**) Phosphorylation reactions are represented with black arrows and dephosphorylation reactions by grey arrows. The name of the yeast enzyme (when relevant) is written on top of its human homologue.

Membranes are composed of two phospholipid leaflets organized as a bilayer in which sterols, glycolipids and proteins are inserted. The phospholipids of this bilayer are amphiphilic with a hydrophilic group (head) linked to a hydrophobic group (tail) (Figure 1A). In the bilayer, the hydrophobic groups face each other, thus creating a hydrophobic space in between them, which ensures its role as a barrier. This property is very important for the anchoring of hydrophobic molecules, such as sterols or ceramides, transmembrane domains or the lipid anchor of proteins. The lipid composition of membranes varies according to the organism (eukaryotes or prokaryotes), the cell type (among the different tissues of a multicellular organism), the membrane type (plasma membrane, endoplasmic reticulum, endosomes, Golgi and other intracellular compartments) or even the state of the cell (quiescent or responding to stress or stimuli) [1,2].

Membranes are mainly composed of five phospholipids: phosphatidylcholine (PC), phosphatidylethanolamine (PE), phosphatidylserine (PS), phosphatidylinositol (PtdIns) and sphingomyelin (SM). Sterols modulate the fluidity of the membrane, which is essential for the lateral diffusion of molecules in the bilayer. The lipid bilayer of membranes has also an asymmetric phospholipid composition between the inner and the outer leaflet, which partly results from the vertical diffusion between the two leaflets by a flip-flop mechanism with a low intrinsic rate compensated by the presence of proteins called flippases [3,4].

If the plasma membrane plays an essential role as a selective barrier, there are many more intracellular membrane structures in eukaryotic cells, such as organelles and transport vesicles. Both the organization, as well as the composition of these membranes depends on the nature of the compartment. Indeed, the endoplasmic reticulum, the Golgi, lysosomes (vacuole in yeast), endosomes and transport vesicles are surrounded by a single lipid bilayer. The inner space of these intracellular compartments is called the lumen. On the other hand, the nucleus, mitochondria, chloroplasts and autophagosomes are structures with at least a double lipid bilayer. Each of these compartments performs specific functions necessary for the life of the cell [1]. Indeed, the vesicular transport of proteins between compartments is regulated in a spatiotemporal manner. It is therefore vital that the cell discriminates between compartments. This relies on the attribution of a specific identity to each organelle and sometimes even to each face of an organelle, such as the *cis* and *trans* faces of the Golgi apparatus. These identity cards are essentially defined by molecules present on the cytoplasmic leaflet of membranes. Among these molecules, membrane lipids and more specifically phosphoinositides are key players.

### 1.2. Phosphoinositides, Lipid Signaling Molecules

Phosphoinositide is a term used to describe the seven types of phosphorylated phosphatidylinositol (PtdIns). Here, we use the abbreviation PPIn for phosphoinositides, since this term is less ambiguous as PtdIns(s) or phosphoinositides (PIs). This PPIn term was first introduced by Robert H. Michell and colleagues in a review [5]. Phosphoinositides (PPIn) are minor constituents of cellular membranes, representing about 1% of total cellular phospholipids with phosphatidylinositol representing about 10% [6]. PPIn are composed of glycerol esterified in positions SN1 and SN2 by two fatty acid chains and linked in position SN3 to an inositol ring by a phosphate group (Figure 1A) [6]. In human, the most common fatty acids in PtdIns are stearic acid (18:0) in position SN1 and arachidonic acid (20:4) in SN2 [7]. In the yeast *Saccharomyces cerevisiae* (*S. cerevisiae*), the nature of the fatty acids is different with palmitic acid (16:0) in position SN1 and oleic (18:1) or palmitoleic acid (16:1) in SN2 [8].

The inositol ring of PPIn is a polyol cyclohexane of which positions D3, D4 and D5 can be phosphorylated, generating seven possible PPIn (Figure 1B): phosphatidylinositol 3-phosphate (PtdIns3*P*), PtdIns4*P*, PtdIns5*P*, PtdIns 3,4-bisphosphate (PtdIns(3,4)*P_2_*), PtdIns(3,5)*P_2_*, PtdIns(4,5)*P_2_* and PtdIns 3,4,5-trisphosphate (PtdIns(3,4,5)*P_3_*). Despite their low concentration in membranes, PPIn play an essential role in the recruitment and/or activation of effector proteins. Moreover, their presence in a given membrane and their levels are determined by lipid kinases and phosphatases specific to the different membranes, allowing the spatiotemporal regulation of various events, such as budding, membrane fusion and dynamics [6].

### 1.3. Phosphatidylinositol, the Precursor of Phosphoinositides

PtdIns, the starting point of the PPIn metabolism, is a ubiquitous phospholipid in eukaryotic cells present in various proportions according to the type of membrane. Indeed, PPIn are all metabolized directly or sequentially from PtdIns (Figure 1). In *S. cerevisiae*, PtdIns is synthesized by the PtdIns synthase 1 (Pis1) on the cytoplasmic face of the Endoplasmic Reticulum (ER), the Golgi, mitochondria and microsomes [9]. In human, its synthesis is catalyzed by a homologue of Pis1, the PtdIns synthase with similar cell distribution as the yeast Pis1 [10].

PtdIns is also recycled by the conversion of PtdIns3*P*, PtdIns4*P* and PtdIns5*P* through the action of the corresponding lipid phosphatase (Figure 1B). In human, myotubularin MTM1 and myotubularin-related phosphatases MTMR1-4, MTMR6 and MTMR7 are 3-phosphatases dephosphorylating specifically the D3 position, thus producing PtdIns from PtdIns3*P* [11]. Less specific phosphatases, such as Sac1, can dephosphorylate PtdIns3*P* in PtdIns, but also PtdIns4*P* and probably PtdIns5*P* in PtdIns [12]. In *S. cerevisiae*, there is only one D3 specific phosphatase called Ymr1. It shares this activity with other phosphatases, synaptojanin-like proteins (Sjl2 and Sjl3) and Sac1, which also convert PtdIns4*P* in PtdIns [13].

## 2. PtdIns4P a Key Trafficking Effector for Phospholipids and Sterols

### 2.1. PtdIns4P Synthesis

PtdIns4P accounts for about 30% of total PPIn in yeast and approximately 45% in Human [6]. It is enriched at the Golgi where it is mainly produced by the phosphorylation of PtdIns by PtdIns 4-kinases [14]. It is also obtained after the dephosphorylation of PtdIns(4,5)P2 and PtdIns(3,4)P2 by PtdIns 5-phosphatases and PtdIns 3-phosphatases respectively (Figure 1B). In S. cerevisiae, the PtdIns 4-kinases Pik1 and Stt4 convert PtdIns in PtdIns4P at the Golgi and the plasma membrane respectively [15]. Dephosphorylation of PtdIns(4,5)P2 in PtdIns4P is performed by the 5-phosphatases Inp51/Sjl1, Inp52/Sjl2, Inp53/Sjl3 and Inp54 (Figure 1B). In Human, PPIn 4-kinases PI4Kα and PI4Kβ synthesize PtdIns4P from the precursor PtdIns. Moreover, PtdIns4P is synthesized from PtdIns(3,4)P2 by the 3-phosphatase PTEN or from PtdIns(4,5)P2 by the 5-phosphatases OCRL1 (Occulocerebrorenal syndrome protein 1), INPP5B (Inositol Polyphosphate Phosphatase 5B), INPP5E and synaptojanines 1 et 2 (Figure 1) [16]. Mutations affecting many of these lipid phosphatases are linked to diseases such as Cowden and cancer for PTEN [17], Lowe syndrome for the OCRL1 [18], Joubert and MORM syndromes (two ciliopathies) for the INPP5E [19,20] and Parkinson’s disease for the Synaptojanin 1 [21,22].

### 2.2. Physiological Role of PtdIns4P

PtdIns4*P* was for a long time merely considered as a precursor of other PPIn. In yeast, PtdIns4*P* is present in two distinct compartments: the plasma membrane and the Golgi [15]. The Golgi is a central node in membrane trafficking, where proteins and lipids from various intracellular compartments get exchanged, thus requiring a tight spatiotemporal regulation of their sorting. PtdIns4*P* is thought to be involved in Golgi trafficking [23]. Hence, in yeast, PtdIns4*P* was shown to have a function in the anterograde transport from the trans-Golgi and the retrograde transport from the Golgi to the endoplasmic reticulum [15]. In the anterograde transport, PtdIns4*P* is required for the formation of secretory vesicles emanating from the Golgi and targeted to the plasma membrane [24]. Many proteins interacting with PtdIns4*P* have been identified and localized at the Golgi [25]. Among them, seven oxysterol binding protein homologues, termed Osh1 to Osh7, have been described, and most of them were shown to transport lipids in association with PtdIns4*P*. Osh1, required for the transport of sterols, localizes to the Golgi through its PH domain (pleckstrin homology), which interacts with PtdIns4*P* [26]. Osh4 extracts and transports ergosterol from the ER membrane to the Golgi, where it substitutes this sterol for PtdIns4*P*, which is in turn transported backward from the Golgi to the ER [27]. Osh6 is a phosphatidylserine (PS) phospholipid transporter that extracts PtdIns4*P* from the plasma membrane (PM) to the ER where it is exchanged for PS to mediate its transport from the ER to the PM. At the ER, Sac1 dephosphorylates PtdIns4*P* to maintain the PtdIns4*P* gradient driving this transport of PS [28].

The human OSBP (oxysterol binding protein) protein and the 12 OSBP-related proteins (ORP in the human genome) are also involved in the intracellular transport of lipids in association with PtdIns4*P*. OSBP localizes to the Golgi through the interaction of its PH domain with PtdIns4*P*. However, this Golgi localization also requires a Golgi GTPase (ADP-ribosylation factor or ARF) [26]. OSBP exchanges sterols for PtdIns4*P* between the ER and the Golgi, and at the ER, PtdIns4*P* is hydrolyzed in PtdIns by the SAC1 phosphatase; the energy produced is further used for sterol transfer [29]. ORP5 and ORP8, involved in PS transport between the ER and the plasma membrane, are localized to ER-PM contact sites via the interaction of their PH (Pleckstrin Homology domain) domain with PtdIns4*P* [30]. ORP5 and ORP8 also localize to contact sites between the ER membrane and the mitochondria and are required for mitochondrial functions [31]. The VAP proteins (VAPA and VAPB) are anchored at the ER membrane, and their absence leads to a significant increase in the endosomal level of PtdIns4*P* due to the impaired OSBP function (and probably other ORPs), a direct binding partner of VAP [32]. This lack of VAP proteins results in dysfunction of the endosome to Golgi membrane trafficking via the retromer complex and the accumulation of actin comets between the endosome and the Golgi involving the endosomal WASH (Wiskott–Aldrich syndrome homologue) complex [32]. FAPP1 and FAPP2 (four-phosphate-adapter proteins 1 and 2) proteins, required for glycosphingolipid metabolism, form a complex with ARFs at the *Trans*-Golgi Network (TGN) and interact with PtdIns4*P* through their PH domain. They are involved in membrane sensing processes [25,33]. PI4Kα also produces PtdIns4*P* at endosomes, the synthesis of which is required for receptor sorting at the early endosomes, maybe via the EH Domain Containing 3 (EHD3) protein [34].

Recent studies on the primary cilium, an antennae projecting from the surface of the cell and whose function or formation is altered in human diseases termed ciliopathies, show that they are enriched in PtdIns4*P*, whereas PtdIns(4,5)*P*_2_ is not detectable. However, upon inactivation of the phosphoinositide 4-phosphatase INPP5E (observed in ciliopathies due to mutations in *INPP5E*), its substrate the PtdIns(4,5)*P*_2_ (enriched at the plasma membrane) is detected at the ciliary tip where it recruits its downstream effector TUL3P, which alters the Sonic hedgehog signaling (Shh) pathway [35].

## 3. Phosphatidylinositol 3-Phosphate (PtdIns3*P*) an Endosomal Lipid Essential in Membrane Trafficking and Autophagy

### 3.1. PtdIns3P Synthesis

PtdIns3*P* accounts for about 30% of total PPIn in yeast and is as abundant as PtdIns4*P*. In human cells, it represents less than 15% of monophosphorylated PPIn and is much less abundant than PtdIns4*P* [6]. PtdIns3*P* is produced by the phosphorylation of PtdIns at position D3 of inositol or by the dephosphorylation of PtdIns(3,4)*P*_2_ or PtdIns(3,5)*P*_2_ (Figure 1).

In *S. cerevisiae*, a single enzyme, the lipid kinase Vps34 (vacuolar protein sorting 34), catalyzes specifically the phosphorylation of PtdIns in PtdIns3*P* [36,37]. The Vps34 kinase is activated via its binding to the protein kinase Vps15, which is anchored to membranes. This Vps34-Vps15 complex further recruits additional effectors to regulate either endosomal trafficking (via Vps38-Vps30 proteins) or autophagy (via Atg14-Vps30) [38]. Interestingly, it has been shown that the positive regulation of Vps34 by Vps15 is stimulated by the direct interaction between the C-terminal region of Vps15 constituted by seven repeats of the WD (Trp-Asp) domains and the Gα subunit Gpa1. Thus, this C-terminal domain could behave as a γ subunit of the yeast G protein. This would result in a coupling between the G protein mediated signaling at the plasma membrane and receptor sorting at the endosomes. Moreover, the interaction of the Vps34-Vps15 complex with Gpa1 stimulates PtdIns3*P* production on endosomes [39].

The human genome encodes eight lipid kinases able to phosphorylate PtdIns at the D3 position, which are classified into three categories according to their substrate specificity and homology [40,41]:

- Two members of the class I phosphoinositide 3-kinases (PI3KC), which phosphorylate predominantly PtdIns(4,5)*P*_2_ to generate PtdIns(3,4,5)*P*_3_
- Three members of the class II phosphoinositide 3-kinases (PIK3C2), which predominantly phosphorylate PtdIns4*P* to generate PtdIns(3,4)*P*_2_
- One member of the class III phosphoinositide 3-kinase (homologous to the yeast Vps34 lipid kinase). As Vps34, the human PIK3C3 is specific for PtdIns and is consequently most probably the biggest source of cellular PtdIns3*P*. The regulatory subunit of VPS34/PIK3C3 is the protein p150/PIK3R4, the homologue of the yeast Vps15 [42]. A phylogenetic study suggests the co-evolution of VPS34/PIK3C3 and its regulatory subunit VPS15/PIK3R4 in most eukaryotes, from yeast to human and plants and in many protists, such as amoeba [43]. However, a recent study shows that in human cells, VPS15 has an additional function in trafficking from Golgi to primary cilia, independent from its association with VPS34, and that a missense mutation in the *VPS15* gene is responsible for ciliopathy [44].

In yeast, PtdIns3*P* is also synthesized by the lipid phosphatases Fig4 (factor induced gene 4, also known as Sac3), Sjl2/Inp52, Sjl3/Inp53 and Sac1. They all possess a SAC catalytic domain able to dephosphorylate PtdIns(3,5)*P*_2_ in PtdIns3*P* [16]. Fig4 is the sole PtdIns 5-phosphatase specific for PtdIns(3,5)*P*_2_ [45]. Its human homologue FIG4/SAC3 fulfills similar cellular functions. Mutations in the human *FIG4* gene cause Charcot-Marie-Tooth type 4J disease, a recessive neuromuscular disease characterized by neuron demyelination [16]. With *MTM1*, *MTMR2*, *MTMR5* and *MTMR13*, *FIG4* is thus the fifth gene encoding a lipid phosphatase involved in a neuromuscular disease [46]. Indeed, mutations in the gene coding for the lipid phosphatase MTM1 cause X-linked myotubular myopathy (XLMTM), also known as centronuclear myopathy, likely by deregulating PtdIns3*P* metabolism [47]. Indeed, it was shown that the disruption of the PIK3C2B kinase resulted in a complete prevention of the myopathy phenotypes in a *Mtm1* disease mouse model, and inhibition of the PIK3C2B kinase activity after appearance of myopathy symptoms promoted a striking rescue in the zebrafish model [48].

### 3.2. Physiological Role of PtdIns3P

In the yeast *S. cerevisiae*, the deletion of the *VPS34* gene is not lethal, but results in the very slow growth of cells and reduced resistance to numerous stress factors (temperature, pH, ethanol, hygromycin B, hyperosmotic stress, caffeine or rapamycin). The yeast *vps34Δ* mutant displays also strong membrane trafficking defects, which result in an altered cell morphology, the defective vacuolar transport of carboxypeptidase Y (CPY) and the absence of autophagy [38]. One of the essential roles of Vps34 in trafficking is to ensure proper sorting of proteins to the vacuole by producing PtdIns3*P*, the latter allowing the recruitment of endosomal effector proteins [49]. PtdIns3*P* production by Vps34 is also critical for autophagy, since it regulates autophagosome formation [50]; the PROPPIN Atg18 and Hsv2 proteins, which contain two binding sites for PtdIns3*P*, are critical for this process [51].

In yeast and mammalian cells, PtdIns3*P* is enriched at endosomal membranes and membranes of intraluminal vesicles of the multivesicular body (MVB) [52]. At the early endosomes, it plays a central role in recruiting effector proteins, such as the yeast Vps27 protein or its human homologue Hrs (hepatocyte growth factor-regulated tyrosine kinase substrate), subunits of the ESCRT-0 (endosomal sorting complex required for transport) complex are involved in the endosomal sorting of proteins and the formation of the MVB [49]. They both have a FYVE domain (Fab1, YGL023, Vps27 and EEA1), which interacts with endosomal PtdIns3*P* [53]. Similarly, the PtdIns3*P* 5-kinase Fab1 (*S. cerevisiae*) or its human homologue PIKfyve, catalyzes the phosphorylation of PtdIns3*P* in PtdIns(3,5)*P*_2_, after their interaction with PtdIns3*P* mediated by a FYVE domain [6]. Hence, one of the physiological roles of PtdIns3*P* is to serve as a precursor for PtdIns(3,5)*P*_2_.

The human adapter protein EEA1 (early endosomal antigen 1) has a high affinity for PtdIns3*P* through its FYVE domain and regulates endosomal membrane fusion processes by recruiting the GTPase Rab5 to endosomes [53].

However, studies show that Hrs is not required for MVB formation, but rather for lysosomal targeting and that it is the sorting nexin SNX3 that is required for MVB formation via binding to PtdIns3*P* through its Phox-homology PX domain [54,55]. Indeed, the PX domain was shown to preferentially bind to PtdIns3*P*, and these domains are found in the sorting nexin SNX proteins [56]. The SNX proteins are involved in endosomal sorting; some belong to the retromer complex (SNX1, SNX2, SNX5 and SNX6) that allows the transport of proteins from the endosomes to the trans-Golgi network, and some others (SNX4, SNX7 and SNX30) are involved in recycling from the endosomes to the plasma membrane [57].

In human, upon autophagy induction, a protein complex comprising VPS34, VPS15, Beclin1 and ATG14 is formed at ER membranes, where the lipid kinase activity of VPS34 leads to the formation of PtdIns3*P*-enriched regions [58]. The latter then serve as platforms to recruit the double FYVE domain-containing protein 1 (DFCP1) and the WD-repeat protein interacting with phosphoinositides (WIPI) proteins, which are homologous to the yeast Atg18 [59,60,61]. DFCP1 interaction with PtdIns3*P* promotes its translocation to an ER-localized punctate compartment enriched in PtdIns3*P* and autophagosomal proteins. This structure, later termed omegasome by Polson and colleagues, initiates the autophagosome formation [59]. WIPI 1 and 2 are PtdIns3*P* effectors, and their absence leads to the accumulation of omegasome (PtdIns3*P*-positive ER structures) [60]. Moreover, WIPI 1 and 2 are essential for the recruitment of the downstream autophagy partners Atg12, Atg5 and Atg16L and, thus, for the subsequent steps of the autophagy process [62]. Indeed, mutations affecting the binding of human WIPI proteins to PtdIns3*P* have been observed in cancers and neurodegenerative diseases, indicating an important biological role of PtdIns3*P* in the regulation of autophagy and beyond [61].

Moreover, PtdIns3*P* patches formed at the ER membrane by Vps34/PIK3C3 in human cells also serve as a scaffold for the formation of the double membrane enclosed vesicles needed for hepatitis C (HCV) virus genome replication. Indeed, whereas the complete viral cycle of HCV takes place in the cytoplasm, the genome replication is done in double membrane vesicles that originate from the cup-like structures built after the interaction of PtdIns3*P* with DFCP1, as do autophagosomes [63].

## 4. PtdIns5*P*, an Underappreciated Phosphoinositide

### 4.1. PtdIns5P Synthesis

PtdIns5*P* is the most recently identified monophosphorylated PPIn [64]. It was underappreciated for a long time due to its very low basal concentration in mammalian cells, but also due to technical difficulties in separating it from PtdIns4*P*. These technical issues were partly alleviated by new methods used to detect PtdIns5*P* [65]. In basal mammalian cell conditions, PtdIns5*P* represents less than 10% of monophosphorylated PPIn [6]. In humans, PtdIns5*P* can be produced directly from PtdIns by the PIKfyve lipid kinase or by dephosphorylation of PtdIns(3,5)*P*_2_ by myotubularin 3-phosphatases [43,66,67,68,69] (Figure 1B). Cells expressing the oncogenic NPM-ALK (nucleophosmin (ribosome biogenesis regulator) and anaplastic lymphoma kinase (tyrosine kinase receptor)) fusion have high levels of PtdIns5*P* produced by PIKfyve [67] (Bergalet et al., 2015). In vivo, PIKfyve overexpression results in an increase in PtdIns5*P*, whereas PIKfyve^+/null^ heterozygous mice have lower PtdIns5*P* levels compared to control mice, without displaying a negative effect on mice viability [68]. The major source of PtdIns5*P* in human cells comes from the action of the myotubularins, a family of phosphoinositide 3-phosphatases associated to different diseases: X-linked centronuclear myopathy (MTM1), Charcot-Marie-Tooth CMT4B1 (MTMR2), CMT4B2 (MTMR13) and CMT4B3 (MTMR5) [46,70]. In human cell cultures and in myotubes, the founder member of this family, named myotubularin MTM1, dephosphorylates PtdIns(3,5)*P*_2_ in PtdIns5*P* [71]. The myotubularin-related phosphatase MTMR3 in coordination with the PIKfyve kinase produces PtdIns5*P* in response to FGF-1 (fibroblast growth factor) in *Drosophila* fibroblasts, which is required to stimulate cell migration [69]. In human, high levels of PtdIns5*P* are also produced from plasma membrane PtdIns(4,5)*P*_2_ by the bacterial IpgD 4-phosphatase upon infection by *Shigella flexneri*, the causative agent for dysentery [72].

This PPIn seems to be specific for Metazoa, since no PtdIns5P has been detected in wild-type *S. cerevisiae* strains. This is consistent with the fact that Ymr1, the unique yeast myotubularin, does not have any 3-phosphatase activity towards PtdIns(3,5)*P*_2_ [13,73].

### 4.2. Physiological Role of PtdIns5P

The role of PtdIns5*P* in mammalian cells is still poorly understood, and expression of bacteria IpgD is the best means so far to address the physiological roles of PtdIns5*P*. Indeed, initially, the first function of PtdIns5*P* in human cells was discovered after observing the plasma membrane rearrangement and actin cytoskeleton reorganization induced by the expression of IpgD [72]. A fraction of PtdIns5*P* is also found in the nucleus, where it could be involved in stress response in particular by modulating the transcriptional activity of chromatin regulator ING2. ING2 is the first protein in which the PHD finger (Plant HomeoDomain), a zinc finger domain, has been shown to specifically bind PtdIns5*P* [74]. The PH domain of Dok (downstream of tyrosine kinase) proteins also shows a strong binding to PtdIns5*P*, and this binding activates the phosphorylation of Dok proteins in T cell signaling [75]. Moreover, PtdIns5*P* was also shown to be involved in plasma membrane and endosomal functions. At the plasma membrane, elevation of intracellular levels of PtdIns5*P* activates the Rho GTPase Rac1 by specifically binding to the guanine nucleotide exchange factor (GEF) Tiam1, via its C-terminal PH domain, which drives actin remodeling [76]. The endosomal adapter TOM1 interacts directly with PtdIns5*P*, and this interaction delays the endosomal internalization of the EGF receptor, showing that PtdIns5*P* is a regulator of endosomal protein sorting [77]. The addition of PtdIns5*P* to cells increases the number of autophagosomes and PtdIns5*P* sustains autophagy in cells treated with the VPS34 inhibitor wortmannin or knocked down for VPS34, thus in cells depleted for PtdIns3*P* [78].

## 5. PtdIns(4,5)*P_2_*, a Phosphoinositide Involved in Actin Dynamics and in Endocytosis

### 5.1. PtdIns(4,5)P_2_ Synthesis

In yeast, PtdIns(4,5)*P*_2_ is as abundant as PtdIns4*P*, that is about 30% of total PPIn and 90% of the different bisphosphorylated PPIn. In human, it represents about 45% of total PPIn and more than 90% of the different bisphosphorylated PPIn. PtdIns(4,5)*P*_2_ is thus the most abundant PPIn [79].

**Figure 2:**
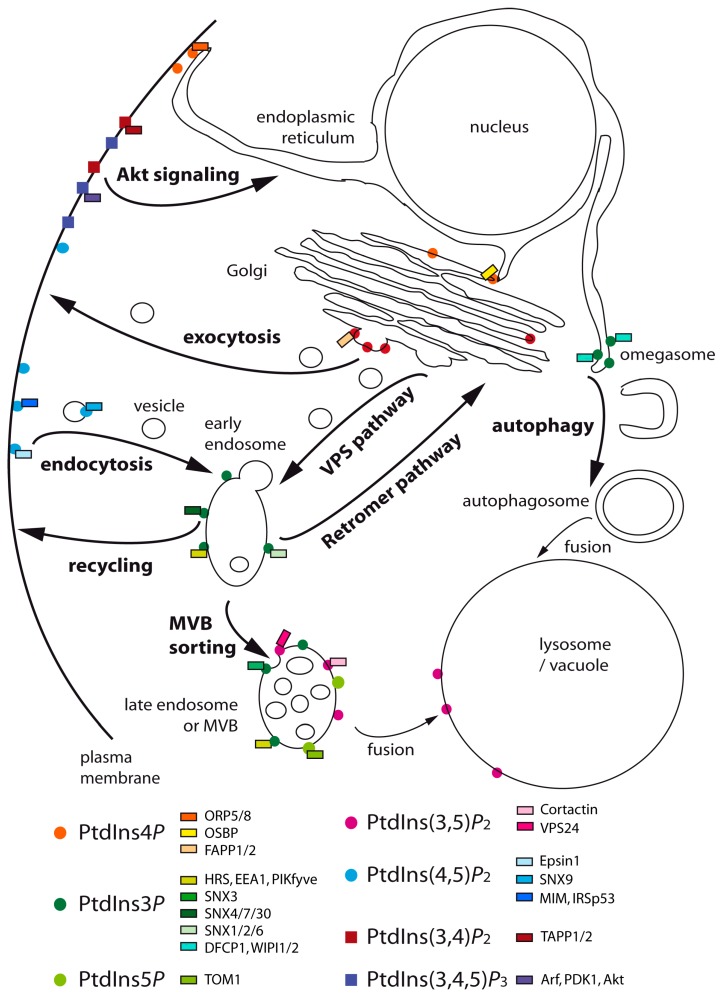
Intracellular localization of the different phosphoinositides and the membrane trafficking pathways. The different phosphoinositides (PPIn) are represented by symbols: circles for PPIn involved in intracellular trafficking with the corresponding steps they regulate; squares for PPIn involved in cell signaling, the latter being absent from yeast. The human proteins interacting with the different PPIn are represented by a rectangle. The MVB stands for multivesicular body and the VPS for vacuolar protein sorting.

PtdIns(4,5)*P*_2_ is synthesized by the PPIn 5-kinase Mss4 in yeast (Figure 1) and is mainly localized at the plasma membrane (Figure 2) [80].

In human, many PPIn-kinases synthesizing PtdIns(4,5)*P*_2_ have been identified: type I PIP5Kα, β and γ are localized at the plasma membrane and convert PtdIns4*P* into PtdIns(4,5)*P*_2_ [81], whereas type II PIP4K are localized at the Golgi and convert PtdIns5*P* into PtdIns(4,5)*P*_2_ [82,83]. The type I PIP5Kβ forms homo- and hetero-dimers with the PIP5Kγ in vitro and in vivo in human cells, and this dimerization is essential for enzymatic activity and plasma membrane localization [84]. In mammalian cells, but not in yeast, PtdIns(4,5)*P*_2_ is also produced from PtdIns(3,4,5)*P*_3_ by dephosphorylation by PPIn 3-phosphatases, such as PTEN (phosphatase and tensin homolog), TPIPα, β and γ (Figure 1) [16].

### 5.2. Physiological Role of PtdIns(4,5)P_2_

In *S. cerevisiae* and in human cells, PtdIns(4,5)*P*_2_ is most abundantly present in the cytoplasmic leaflet of the plasma membrane (Figure 2), where it acts as a major actin cytoskeleton and endocytic regulator. In *S. cerevisiae*, Mss4 acts in combination with the PtdIns 4-kinase Stt4 at the plasma membrane to generate PtdIns(4,5)*P*_2_ from PtdIns. This PPIn is essential for the activation of the MAP kinase Rho1/Pkc1-mediated signaling cascade. Indeed, proper localization of Rom2, the GEF of the Rho1 GTPase, depends directly on the interaction of its PH domain with membrane PtdIns(4,5)*P*_2_ [85]. Actin cytoskeleton organization depends in large part on this signaling pathway [80]. Mss4 activity and PtdIns(4,5)*P*_2_ synthesis fulfill other important functions in endocytosis, which is actin-dependent in yeast [86]. Indeed, effector proteins of endocytosis, such as those with an ENTH (Epsin N-terminal homology), ANTH (AP-180 N-terminal homology) or PH (Pleckstrin homology) domain all interact with PtdIns(4,5)*P*_2_ [87,88]. Among them, the yeast rhomboid protein Rbd2 directly binds PtdIns(4,5)*P*_2_ to regulate and control clathrin-mediated actin-dependent endocytosis [89]. Rbd2 acts in coordination with the syndapin-like protein Bzz1 to control type I myosin-dependent actin polymerization at the sites of endocytosis [89,90].

In mammalian cells, PtdIns(4,5)*P*_2_ is mainly required at the plasma membrane for the actin regulation of clathrin-mediated endocytosis (Figure 2) [79]. Different effector proteins required for the internalization step of endocytosis have an ENTH, ANTH or PH domain, which interacts specifically with PtdIns(4,5)*P*_2_ [79,87,88]. For example, the ENTH domain of Epsin1 interacts with PtdIns(4,5)*P*_2_, inducing a structural rearrangement resulting in the formation of an additional N-terminal α-helix that inserts into the lipid bilayer and generates the membrane curvature required for the formation of endocytic vesicles [91]. The SNX5 and SNX9 sorting nexins preferentially bind PtdIns(4,5)*P*_2_ over PtdIns3*P* and have a membrane remodeling BAR (Bin-amphiphysin-Rvs) domain at their N-terminus [56,92,93]. At the plasma membrane, SNX9 directly binds to the 5-phosphatase OCRL-1, which acts in the late stages of endocytic internalization to uncoat the vesicles and allow cargo sorting by fusion of the vesicles with the endosomes [94]. At the endosomes of non-neuronal cells, PtdIns(4,5)*P*_2_ signaling is turned off by the 5-phosphatase OCRL-1 under the spatiotemporal control of the Rab35 GTPase [95]. P SNX5 is localized on early endosomes and specifically recruited at the plasma membrane in response to EGF stimulation [96]. As in yeast, PtdIns(4,5)*P*_2_ also regulates actin cytoskeleton dynamics. Indeed, several studies have shown the interaction of PtdIns(4,5)*P*_2_ with actin polymerization regulators [97]. At the plasma membrane, two actin-binding proteins MIM (Missing In Metastasis) and IRSp53 (Insulin Receptor Substrate Protein of 53 kDa) directly interact with PtdIns(4,5)*P*_2_ to deform the membrane in coordination with actin cytoskeleton reorganization to induce filopodia and promote cell motility [98]. Actin dynamics is regulated by different small G-proteins (Rho, Rac, Cdc42, Arf) whose activity is modulated by PtdIns(4,5)*P*_2_ [99].

PtdIns(4,5)*P*_2_ has been detected in the nucleus, where it regulates pre-mRNA splicing [100]. Moreover, in the nucleus, PtdIns(4,5)*P*_2_ regulates the activity of a poly(A) polymerase, termed Star-PAP (nuclear speckle targeted PIPKIalpha regulated-poly(A) polymerase) to selectively control the expression of some targeted genes [101]. In humans, PtdIns(4,5)*P*_2_ plays also a major role in regulating various signaling pathways, namely because of the rapid switching it can undergo thanks to the different PPIn kinases and phosphatases using it as a substrate (Figure 1). Highly metastatic breast cancer cells have reduced PtdIns(4,5)*P*_2_ levels at the plasma membrane, mainly due to upregulation of enzymes using it as a substrate [102].

## 6. PtdIns(3,5)*P_2_*, a Regulator of Endosome-Lysosome Trafficking

### 6.1. PtdIns(3,5)P_2_ Synthesis

PtdIns(3,5)*P*_2_ is a rare PPIn, since it represents less than 5% of total PPIn in *S. cerevisiae* and human and is enriched in vesicular and tubular domains in late endosomes and at the vacuole (yeast)/lysosome (HeLa cells) (Figure 2) [79,103].

In *S. cerevisiae*, PtdIns(3,5)*P*_2_ is sequentially synthesized by the PtdIns 3-kinase Vps34 from PtdIns to generate PtdIns3*P*, which is then phosphorylated by the PtdIns3P 5-kinase Fab1 to generate PtdIns(3,5)*P*_2_ (Figure 1). In response to osmotic stresses, PtdIns(3,5)*P*_2_ synthesis is stimulated, and its intracellular level increases 20-fold compared to non-stressed cells [104]. The vacuolar membrane proteins Vac7 and Vac14 are required for PtdIns(3,5)*P*_2_ synthesis [105]. Vac7 is the main activator of Fab1 in response to osmotic shock, whereas Vac14 acts in a complex with the lipid phosphatase Fig4 to regulate PtdIns(3,5)*P*_2_ renewal [106]. This is rather surprising, because stimulating PtdIns(3,5)*P*_2_ synthesis in response to osmotic stress requires two antagonistic processes, PtdIns(3)*P* phosphorylation in PtdIns(3,5)*P_2_*, as well as its dephosphorylation in PtdIns(3)*P* in a seemingly futile cycle [107]. This shows an essential interdependence between lipid kinases and phosphatases in the regulation of PtdIns(3,5)*P*_2_ synthesis.

In human, PtdIns(3,5)*P*_2_ synthesis is similar to yeast, and it is catalyzed by PIKfyve, the sole PtdIns3P 5-kinase (Figure 1B) [108]. Up to now, no PPIn 4-phosphatase hydrolyzing PtdIns(3,4,5)*P*_3_ has been characterized. Thus, the only pathway to synthesize PtdIns(3,5)*P*_2_ requires the phosphorylation of PtdIns3*P* (Figure 1). Furthermore, the PtdIns(3,5)*P*_2_ synthesis pathway described in yeast is conserved in human, with PIKfyve interacting with multiple partners [108]. This interaction is indirect and requires the adapter protein ArPIKfyve/VAC14 to stabilize the complex and stimulate PIKfyve activity [109]. As in yeast cells, VAC14 serves as a platform regulating PtdIns(3,5)*P*_2_ synthesis by interacting directly with PIKfyve, FIG4/SAC3 and VAC7 to fine tune the regulation of PtdIns(3,5)*P_2_* levels [109,110]. Mutation in the *FIG4* gene causes Charcot-Marie-Tooth neuropathy [111], and the *Mtmr2* knock-out mice model for the disease has elevated PtdIns(3,5)*P_2_* levels [112]. Moreover, there is a genetic interaction between MTMR2 that dephosphorylates PtdIns(3,5)*P*_2_ and FIG4 that participates in PtdIns(3,5)*P*_2_ synthesis since reduction of FIG4 rescues the myelin outfoldings phenotype of the *Mtmr2* KO mice [112]. Autosomal dominant mutations in FIG4 are also a rare cause of amyotrophic lateral sclerosis and primary lateral sclerosis [113]. Biallelic mutations in the *VAC14* gene are responsible for neurological disease [114].

### 6.2. Physiological Role of PtdIns(3,5)P_2_

In *S. cerevisiae*, *fab1Δ* cells display a growth phenotype at 23 °C, which are unable to grow at 37 °C, and vacuoles have an acidification defect, as well as being very enlarged, occupying up to 80% of the cell volume. One of the consequences of these enlarged vacuoles is the incorrect segregation of chromosomes during cell division [115]. Vac7 and Vac14, which regulate PtdIns(3,5)*P*_2_ synthesis, are required to maintain the vacuolar morphology, as well as its proper inheritance between the mother and the daughter cell [105].

PtdIns(3,5)*P*_2_ plays an essential role in protein sorting at the late endosomes/MVB (Figure 2) [116]. Membrane proteins destined for the vacuole are tagged with ubiquitin at the endosomes, recognized by ESCRT-0 to -3 complexes for their recruitment at endosomal internalization sites and packaged into the vesicles budding in the endosomal lumen, thus forming the MVB. The fusion of the latter with the vacuole results in the delivering of these vesicles in the vacuolar lumen [49]. The mammalian VPS24 protein, a component of the ESCRT-3 complex, was shown to specifically bind PtdIns(3,5)*P*_2_ [117]. At the endosomes, yeast epsins Ent3 and Ent5 interact with PtdIns(3,5)*P*_2_ through their ENTH domain and are required for endosomal sorting of ubiquitylated cargos and endosomal recycling of SNARES [118,119,120]. To date, the yeast Atg18/Svp1 and Hsv2 proteins, belonging to the PROPPIN family, show the highest affinity and specificity for PtdIns(3,5)*P*_2_; in vitro, these proteins are involved in autophagy [51,121]. These two proteins do also bind to PtdIns3*P* via two distinct sites [51]. Atg18/Svp1 regulates Fab1 activity by interacting with the regulatory protein Vac7 (itself recruited by the scaffold protein Vac14). Consequently, Atg18/Svp1 could act as a PtdIns(3,5)*P*_2_ sensor by feedback regulating of its synthesis through Vac7 and Vac14 [122].

In mouse, *PIKfyve^−/−^* knock-out is embryonically lethal at an early stage [68], underlining the central role of this lipid in cellular processes. PIKfyve was described as having a role in various processes, such as endosomal sorting of proteins, lysosomal homeostasis or signaling pathway regulations [6]. At the endosomes, production of PtdIns(3,5)*P*_2_ induces the release of cortactin from the endosomal branched actin network via direct interaction between the actin filament-binding region of cortactin and PtdIns(3,5)*P*_2_. This regulation is important for membrane trafficking since actin cytoskeleton dynamics regulates membrane curvature and transport of vesicles [123]. At the central nervous system (CNS), PtdIns(3,5)*P*_2_ regulates oligodendrocytes differentiation by directing endosomal trafficking of plasma membrane-derived myelin-associated glycoprotein (MAG) [124].

## 7. PtdIns(3,4)*P_2_*, a Lipid Secondary Messenger

### 7.1. PtdIns(3,4)P_2_ Synthesis

PtdIns(3,4)*P*_2_ is not detected in yeast *S. cerevisiae*. In human cells, it mainly localizes to the plasma membrane (Figure 2) and represents less than 10% of total PPIn in basal conditions. Nonetheless, its intracellular level can transiently increase in response to stimulations by growth factors or cytokines [6]. PtdIns(3,4)*P*_2_ is mainly produced by the phosphorylation of PtdIns4*P* into PtdIns(3,4)*P*_2_ by class II PI3K lipid kinases (Figure 1B) [125]. PtdIns(3,4,5)*P*_3_ dephosphorylation into PtdIns(3,4)*P*_2_ is performed by the PPIn 5-phosphatases SHIP1/INPP5D, SHIP2/INPPL1, OCRL1, INPP5B, as well as synaptojanins 1 and 2 (Figure 1) [16].

### 7.2. Physiological Role of PtdIns(3,4)P_2_

PtdIns(3,4)*P*_2_ acts as a secondary messenger by recruiting the protein kinases Akt (protein kinase B) and PDK1 (phosphoinositide-dependent kinase 1) or Pleckstrin, the downstream effector of protein kinase C (PKC) through their PH domain [126]. A transforming mutation in the PH domain of Akt1 is associated with breast, colorectal and lung cancers; this mutation activates Akt1 by allowing its recruitment to membranes independently of its PtdIns(3,4)*P*_2_ and PtdIns(3,4,5)*P*_3_ binding [127]. The link between PtdIns(3,4)*P*_2_ and the PI3K/Akt signaling pathway suggests that this PPIn could be involved in numerous biological processes, such as controlling the cell cycle, cell survival, angiogenesis or glucose metabolism. The balance between PtdIns(3,4)*P*_2_ and PtdIns(3,4,5)*P*_3_ through the interplay between lipid kinases and phosphatases (Figure 1) is essential in regulating signaling pathways downstream of Akt [128]. The INPP4B phosphatase is acting as a tumor suppressor by dephosphorylating the 4’-position of PtdIns(3,4)*P*_2_ and thus inhibiting the Akt signaling pathway [129].

Among the various protein domains binding PtdIns(3,4)*P*_2_, only the PH domains of TAPP1 (tandem PH domain containing protein 1) interact specifically with PtdIns(3,4)*P*_2_, the specificity of which is due to an alanine residue of the binding domain [130,131]. Double knock-in mice of TAPP1 and TAPP2 affected in the PtdIns(3,4)*P*_2_ binding site display insulin sensitivity and increased activation of Akt, a key player in insulin signaling, showing that in vivo PtdIns(3,4)*P*_2_ is a negative regulator of the PI3K/Akt signaling pathway [132].

## 8. PtdIns(3,4,5)*P_3_*, a Key Effector of the PI3K/Akt Signaling Pathway

### 8.1. PtdIns(3,4,5)P_3_ Synthesis

*S. cerevisiae* does not have detectable levels of PtdIns(3,4,5)*P*_3_, so this PPIn is considered absent from this organism. In human, PtdIns(3,4,5)*P*_3_ represents less than 5% of total PPIn and is almost undetectable in quiescent cells. However, its intracellular level rapidly and transiently increases up to 100-fold in response to an agonist [133]. PtdIns(3,4,5)*P*_3_ is mainly synthesized at the plasma membrane (Figure 2) by class I PPIn 3-kinases from PtdIns(4,5)*P*_2_ (Figure 1), but small pools of PtdIns(3,4,5)*P*_3_ can be found at the membrane of other intracellular compartments in response to agonists [6].

The synthesis of PtdIns(3,4,5)*P*_3_ is tightly regulated given that this signaling molecule is at the center of many signaling pathways. Among the regulators of its intracellular levels, there is the PTEN phosphatase, which catalyzes the dephosphorylation of PtdIns(3,4,5)*P*_3_ on position D3 to produce PtdIns(4,5)*P*_2_. PTEN has also been characterized as a tumor suppressor; indeed, mutations in the *PTEN* gene are linked to many cancers [16]. The main role of PTEN is to regulate the cell cycle and apoptosis through its phosphatase activity, which is required for the regulation of the PI3K/Akt signaling pathway.

### 8.2. Physiological Role of PtdIns(3,4,5)P_3_

Despite being present at very low levels, PtdIns(3,4,5)*P*_3_ is the PPIn with the best characterized cellular functions. Indeed, its effectors are involved in many signaling pathways and interact with it through their PH domain. Among its effectors, we can cite small GTPases of the Arf family (ADP-ribosylation factors), the serine/threonine kinases PDK1 and Akt, as well as phospholipase C γ (PLCγ). Thus, PtdIns(3,4,5)*P*_3_ controls key cellular functions, such as cell proliferation and cell survival, cytoskeleton dynamics, cell motility, membrane trafficking and apoptosis [25,97]. The deregulation of PtdIns(3,4,5)*P*_3_ intracellular levels results in the development of many diseases.

One of the most studied functions of PtdIns(3,4,5)*P*_3_ is the regulation of the Akt kinase. Akt plays a very important, role on the one hand, by activating the class I PI3KC I and PtdIns(3,4,5)*P*_3_ synthesis and, on the other hand, by activating PDK1 at the plasma membrane after its membrane recruitment through the interaction of its PH domain with PtdIns(3,4,5)*P*_3_ [128]. There are three Akt isoforms, Akt1 to Akt3, which are activated by growth factors or other extracellular stimuli, as well as by oncogenic mutations in different regulators of Akt (Ras, subunits p110 and p85 of class I PI3KC and PTEN). Indeed, the PH domain of Akt also interacts with PtdIns(3,4)*P*_2_ produced by SHIP dephosphorylation of PtdIns(3,4,5)*P*_3_ (Figure 1) [128]. Perturbations in the PtdIns(3,4,5)*P*_3_/PtdIns(3,4)*P*_2_/Akt signaling pathway lead to cancers, diabetes and cardiovascular and neurological diseases. Many of the inhibitors/antagonists commonly used in therapies act on the interaction between PPIn and the PH domain of Akt [128]. PtdIns(3,4,5)*P*_3_ also promotes rearrangements of the actin cytoskeleton in response to growth factors via the activation of the Rho GTPase Cdc42 that mediates filamentous actin assembly. This dynamic assembly of actin plays an important role in the translocation of the meiotic spindle from the center of the oocyte to the cortex [97,134].

## 9. Conclusions

Phosphoinositides are lipid molecules coordinating and regulating intracellular trafficking. The synthesis of these different PPIn is temporally and spatially controlled by the interplay between lipid kinases and phosphatases in response to stimuli. The seven potential PPIn lipids are present in most cell types at various levels with most species enriched in a given intracellular compartment. All PPIn are virtually interconvertible as long as the organism has the appropriate set of enzymes for their metabolism. This explains why PtdIns5*P*, PtdIns(3,4)*P*_2_ and PtdIns(3,4,5)*P*_3_, are present in mammalians and have not been detected in the yeast *S. cerevisiae*. PPIn play an essential role in defining the identity of intracellular membranes. Moreover, local changes in PPIn levels allow the fine regulation of key cellular events, such as vesicular budding, membrane fusion or the dynamics of trafficking. Given their low abundance (less than 10% of cellular phospholipids), PPIn can be locally subjected to strong variations of their concentration. This is particularly the case for PtdIns(3,4,5)*P*_3_ in response to stimuli in mammalian cells and for PtdIns(3,5)*P*_2_ in response to an osmotic shock in yeast. Therefore, many studies have been and will have to be done to understand the metabolism of PPIn, their localization and intracellular functions. Indeed, despite their low levels, they play essential roles in the recruitment and/or activation of effector proteins and are involved in the regulation of many cellular functions.
